# A dual-tracer approach using [^11^C]CH and [^18^F]FDG in HCC clinical decision making

**DOI:** 10.1186/s13550-023-01024-y

**Published:** 2023-08-29

**Authors:** Emile B. Veenstra, Simeon J. S. Ruiter, Robbert J. de Haas, Koert P. de Jong, Paola A. Erba, Rudi A. J. O. Dierckx, Walter Noordzij

**Affiliations:** 1 Department of Nuclear Medicine and Molecular Imaging, Medical Imaging Center, University of Groningen, University Medical Center Groningen, P.O. Box 30.001, 9700 RB Groningen, The Netherlands; 2 Department of Hepato-Pancreato-Biliary Surgery and Liver Transplantation, University of Groningen, University Medical Center Groningen, Groningen, the Netherlands; 3 Department of Radiology, Medical Imaging Center, University of Groningen, University Medical Center Groningen, Groningen, the Netherlands; 4 Department of Medicine and Surgery, Nuclear Medicine UnitASST – Ospedale Papa Giovanni, University of Milan-Bicocca, Piazza, Bergamo, Italy

**Keywords:** Dual-tracer PET/CT imaging, Hepatocellular carcinoma, [^18^F]FDG, [^11^C]CH, Clinical decision making

## Abstract

**Background:**

Early detection of recurrent or progressive HCC remains the strongest prognostic factor for survival. Dual tracer PET/CT imaging with [^11^C]CH and [^18^F]FDG can further increase detection rates as both tracers entail different metabolic pathways involved in HCC development. We investigated dual-tracer PET/CT in clinical decision making in patients suspected of recurrent or progressive HCC. All HCC patients who underwent both [^11^C]CH and [^18^F]FDG PET/CT in our institute from February 2018 to December 2021 were included. Both tracer PET/CT were within 4 weeks of each other with at least 6-month follow-up. Patients underwent dual tracer PET/CT because of unexplained and suspicious CT/MRI or sudden rise of serum tumour markers. A detected lesion was considered critical when the finding had prognostic consequences leading to treatment changes.

**Results:**

Nineteen patients who underwent [^11^C]CH and [^18^F]FDG PET/CT were included of which all but six patients were previously treated for HCC. Dual-tracer critical finding detection rate was 95%, with [^18^F]FDG 68%, and [^11^C]CH 84%. Intrahepatic HCC recurrence finding rate was 65% for both tracers. [^18^F]FDG found more ablation site recurrences (4/5) compared to [^11^C]CH (2/5). Only [^11^C]CH found two needle tract metastases. Both tracers found 75% of the positive lymph nodes. Two new primary tumours were found, one by [^18^F]FDG and both by [^11^C]CH.

**Conclusions:**

Our study favours a dual-tracer approach in HCC staging in high-risk patients or when conventional imaging is non-conclusive.

## Background

Hepatocellular carcinoma (HCC) is a major cause of global morbidity and mortality with rising incidence [1]. Therefore, early detection of recurrent or progressive tumour remains the strongest prognostic factor for survival [2, 3]. Currently, diagnostic imaging with dynamic CT and MRI are currently advised in detecting progression of HCC according to ESMO clinical guidelines [4].

The Barcelona clinic liver cancer (BCLC) staging system currently divides therapeutic options for HCC into: curative treatments (surgical resection, ablation, and liver transplantation) in case of very early or early stage HCC (A), transarterial chemoembolization (TACE) or selective internal radiotherapy (SIRT) for intermediate stage HCC (B), sorafenib for advanced stage HCC (C), and best supportive care for terminal stage HCC (D) [5]. Imaging after treatment with dynamic CT or MRI can be complicated by fibrosis and heterogenous residual tumour appearance and extrahepatic metastasis [4, 6]. Histological analysis of tumour differentiation is often not performed, but is a major predictive factor of post-operative recurrence in HCC [7]. This motivated the use PET imaging as non-invasive marker of tumour differentiation with the potential to be a more sensitive method for localizing tumour recurrence [8].

[^18^F]-fluoro-2-deoxy-d-glucose ([^18^F]FDG) PET/CT evaluates tumour viability based on glycolytic activity. Success has been achieved in HCC with sensitivity ranging from 36 to 70% after treatment [9–11]. Less success has been realized for multifocal HCC and non-bone extrahepatic metastases [10, 12]. The sensitivity of [^18^F]FDG PET/CT for well-differentiated intrahepatic HCC is similar to conventional imaging [13–18]. To overcome this lack of sensitivity for certain extrahepatic and intrahepatic metastases, the [^11^C]Choline ([^11^C]CH) tracer of cell membrane lipid metabolism is used. HCC may show a high proliferation and increased metabolism of cell membrane components, which will lead to an increased uptake of choline [15]. Clinical studies of [^11^C]CH show better detection rates than [^18^F]FDG PET/CT for well to moderately differentiated HCC lesions (84%) [11].

As both tracers relate to different metabolic pathways involved in tumour development, it is hypothesized that poorly differentiated HCC could be better evaluated with [^18^F]FDG and well-differentiated HCC with [^11^C]CH [19, 20]. This led to the consideration of dual-tracer imaging in HCC as tumour differentiation of lesions within a given patient may vary, some taking up only one tracer. Variability of uptake can also be seen between separate portions of a single HCC lesion. Due to high background uptake of liver parenchyma of [^18^F]FDG, intrahepatic lesions might more difficult to detect, further complicated by the display of significant treatment effects seen on PET/CT in post-therapy HCC cases [21]. Therefore, this study aims to evaluate the role of dual-tracer PET imaging with [^18^F]FDG and [^11^C]CH PET/CT in diagnosed HCC patients with suspicion of recurrent or progressive HCC in clinical decision making.

## Methods

This case series study included HCC patients who underwent both [^11^C]CH and [^18^F]FDG PET/CT in our institute from February 2018 to December 2021. No exclusion criteria were applied. HCC patients were included if they received both [^11^C]CH and [^18^F]FDG PET/CT within 4 weeks of each other and had at least 6 months of follow-up. Patients underwent both radiotracer PET/CT scanning because intra- and extrahepatic recurrence of HCC was suspected on conventional imaging or patients had elevated serum tumour markers when conventional imaging was inconclusive. Tumour markers were considered elevated it they significantly increased compared with previous tumour marker measurements as part of normal follow-up. As the study was performed retrospectively and both radiotracers are part of clinical routine in our institution, individual informed consent was not required, according to the Dutch Act on Medical Scientific Research involving Human Beings (WMO). All research was conducted in accordance with both the Declarations of Helsinki and Istanbul.

A multidisciplinary team consisting of a hepato-pancreato-biliary surgeon, radiologist, and nuclear medicine physician discussed follow-up status of HCC patients, often with a history of treatment, such as microwave ablation (MWA), selective internal radiotherapy (SIRT) or transarterial chemoembolization (TACE), and surgical resection. These patients underwent dual-tracer PET/CT scanning in cases of unexplained and suspicious CT/MRI anatomical imaging or sudden rise of serum tumour markers without anatomical imaging evidence. General patient characteristics, medical imaging, relevant histopathology, alfa-fetoprotein (AFP) and des-gamma-carboxyprothrombin (DGCP), BCLC stage, follow-up therapy, and biopsy data for primary tumours, not metastases, were extracted from patient medical records.

PET/CT images were examined by author EBV and related to radiologist’s report of both tracer studies and noted whether a lesion was detected by any of the tracers. No case discrepancies were encountered. A lesion was considered malignant if there were non-physiological foci of high uptake, unless the imaging context concluded benign origin. A malignant lesion was considered critical when the finding had prognostic consequences leading to treatment changes. On the contrary, non-critical findings were defined as findings not altering treatment strategy, for example in case of a novel extrahepatic metastasis in patients with already known extrahepatic disease.

### PET/CT and FDG/CHOLINE

All images were taken on a Biograph mCT40 PET/CT (Siemens Healthcare, Erlangen, Germany). Before both PET/CT studies, patients were instructed to fast for at least 6 h. The [^11^C]CH had a scheduled activity of 400 MBq (10.8 mCi), irrespective of body mass. [^11^C]CH or [^18^F]FDG (3 MBq or 0.08 mCi per kg of body mass) administered intravenously in an infusion line connected to saline. Low-dose CT was acquired first, followed by PET acquisition 5 min after [^11^C]CH injection or 60 min after [^18^F]FDG injection, covering a field of view from the skull to mid thighs.

PET data was reconstructed with Siemens Ultra HD (TrueX and time of flight), using 3 iterations and 21 subsets with a 400-matrix size and a 9-mm Gaussian (isotropic) filter. Attenuation and scatter correction of PET emission data were achieved by a low-dose CT scan with 120 kV and 35 mAs. For [^18^F]FDG, SUVmax was determined according to EARL and corrected for blood glucose level.

## Results

Nineteen patients who underwent [^11^C]CH and [^18^F]FDG PET/CT examinations were identified (Table 1). Patients were previously diagnosed with HCC by imaging (n = 10) or by pathology (n = 9). Of these nine patients, histopathologic results of the primary HCC lesion revealed four well-, two moderately-, and three poorly differentiated tumours. All but seven patients were previously treated for HCC, including MWA (n = 6), SIRT (n = 1), surgical resection (n = 1), TACE (n = 1), MWA and resection (n = 2), and MWA and SIRT (n = 1). Six patients had not yet been treated, as they received dual-tracer PET/CT while awaiting planned therapy (four SIRT, two MWA). Other patients were under active surveillance, with no planned treatment. All but two patients had underlying diffuse liver disease: cirrhosis (n = 9) with one case caused by chronic hepatitis B virus and one by hereditary hemochromatosis, fibrosis (n = 1), non-alcoholic steatohepatitis (NASH, n = 6), and steatosis (n = 1). Nine patients were considered BCLC stage A, seven stage B, and three stage C. The minimum length of follow-up was 12 months, with a maximum of 24.

**Table 1:** Patient and clinical characteristics with PET/CT findings

Sex	Age (years)	Scan delay^a^ (days)	Underlying disorder liver	BCLC stage	Previous therapy	Degree of differentiation at histopathology	Reason for dual-tracer examination	Critical finding	18F-FDG	11C-CH	Other findings	Follow-up therapy
M	49	2	NASH	B	MWA	N/A	Suspect CT/MRI	Recurrence ablation site, mediastinal lymph node	+	−	Both: mHCC m. adductor longus	Palliative with chemotherapy
F	71	0	Steatosis	C	MWA, Resection	Poorly	Rise of tumour marker	Recurrence ablation site, precaval lymph node	+	+	CH: needle tract recurrence, prostate carcinoma	BSC
M	72	1	NASH	0	SIRT	Poorly	Suspect CT/MRI	Retrocaval lymph node	+	+		Immunotherapy
M	77	0	None	B		Poorly	Suspect CT/MRI	HCC liver right lobe and portocaval lymph node	+	+	FDG: liver lymph node CH: HCC S1	Start systemic immunotherapy
F	64	0	NASH	B	MWA	Moderately	Suspect CT/MRI	mHCC Th6	+	+		Radiotherapy
F	74	3	Cirrhosis (HBV)	A		Moderately	Rise of tumour marker	Growth known HCC S5/6	−	+		Surgical partial liver resection
F	65	0	Cirrhosis (Alcohol)	B		Well	Suspect CT/MRI	Bilateral lung carcinoma	+	+		Surgical partial lung resection
F	75	0	Fibrosis	B	Resection	Well	Rise of tumour marker	HCC S1	+	+		TACE
F	39	12	None	B		Well	Suspect CT/MRI	Multifocal HCC liver right lobe	+	+		OLT
M	75	12	NASH	C	MWA	Well	Suspect CT/MRI	Needle tract recurrence after ablation	−	+	CH: 5^th^ costovertebral junction	BSC
M	74	0	Cirrhosis (Alcohol)	B	TACE	N/A	Suspect CT	HCC recurrence	−	−		SIRT
M	66	0	NASH	A	MWA	N/A	Suspect CT/MRI	Lymph node lesser omentum and a. hepatica communis	−	+	FDG: mHCC ablation site S4/5	BSC
M	67	0	Cirrhosis (Alcohol)	A	MWA	N/A	Rise of tumour marker	mHCC S1, HCC recurrence ablation site S2/3	+	+	FDG: tumour thrombus v. porta	Palliative with sorafenib
M	81	1	Cirrhosis (Hemochromatosis)	A	SIRT, MWA	N/A	Suspect CT/MRI	Thoracic lymph nodes	+	+	FDG: recurrence ablation site	Palliative with sorafenib
M	60	0	Cirrhosis (Alcohol)	A		N/A	Suspect CT/MRI	Known HCC S7/8	+	+		SIRT
F	76	0	Cirrhosis (Alcohol)	C		N/A	Suspect CT/MRI. Rise of tumour marker	Known HCC S7/8	+	+		SIRT
M	53	3	Cirrhosis (Alcohol)	A	MWA, Resection	N/A	Rise of tumour marker	mHCC pararectal fat	−	+		Surgical local resection
M	57	0	NASH	A		N/A	Rise of tumour marker	mHCC liver subcapsular S8	−	+		MWA
F	62	18	Cirrhosis (Alcohol)	0	MWA	N/A	Rise of tumour marker	HCC S7/8	+	−		MWA

^a^Days between patient receiving one and the other PET/CT scan

*M* male, *F* female, *NASH* non-alcoholic steatohepatitis, *HBV* hepatitis B-virus, *BCLC* Barcelona clinic liver cancer, *MWA* microwave ablation, *SIRT* selective internal radiation therapy, *TACE* trans-arterial chemoembolization, *N/A* not available, *BSC* best supportive care, *OLT* orthotopic liver transplantation

### PET/CT

Eleven patients received both scans on the same day and all patients underwent both scans within 21 days. All patients had been staged before onset of suspicion. In 11 cases inclusion for dual-tracer imaging was due to non-conclusive CT/MRI findings, one patient had both non-conclusive CT/MRI imaging and significant rise of tumour marker, and seven patients were considered for dual-tracer PET/CT only having unexplained rise of tumour markers. [^11^C]CH and [^18^F]FDG PET/CT examinations of the same patient were performed in arbitrary order.

### PET findings

Critical findings were found in 68% (13/19) of all cases by [^18^F]FDG and 84% (16/19) by [^11^C]CH (Table 2). Non-critical findings, which were often recurrent multifocal HCC, were found in 63% by [^18^F]FDG and 50% with [^11^C]CH. Critical finding detection rate for both tracers combined was 95%, as one patient had a new intrahepatic HCC recurrence that remained undetected by both PET tracers. With MRI, this patient was diagnosed with progressive disease of the known HCC lesion, which was subsequently successfully treated with SIRT. This patient only received CT follow-up before dual PET/CT scanning due to patient availability.

**Table 2:** PET tracer detection rates

		18F-FDG	11C-CH
	Detection rate Critical finding^b^	13/19 (68%)	16/19 (84%)
	Detection rate non-critical finding	5/8 (63%)	4/8 (50%)
Differentiation^a^	Well	3/4 (75%)	4/4 (100%)
	Moderately	1/2 (50%)	2/2 (100%)
	Poorly	3/3 100%	3/3 (100%)
Multifocal HCC	Recurrence	10/16 (63%)	11/16 (69%)
	Vascular involvement	1/1 (100%)	0/1 (0%)
	Total	11/17 (65%)	11/17 (65%)
Extrahepatic HCC	Soft tissue	2/4 (50%)	2/4 (50%)
	Bone	1/1 (100%)	1/1 (100%)
	Lymph node	6/8 (75%)	6/8 (75%)
	Total	11/13 (80%)	9/13 (73%)
	New primary tumours	1/2 (50%)	2/2 (100%)
Therapy-related	Needle tract metastases	0/2 (0%)	2/2 (100%)
	Ablation site recurrence	4/5 (80%)	2/5 (40%)
	Total	4/7 (57%)	4/7 (57%)

Combined detection rates were 100% for all lesions, except for one HCC recurrence, which remained undetected by both tracers

^a^Differentiation established according to histopathology

^b^Critical finding means the tracer found a lesion which was integral in consequent clinical decision making

**Fig. 1:**
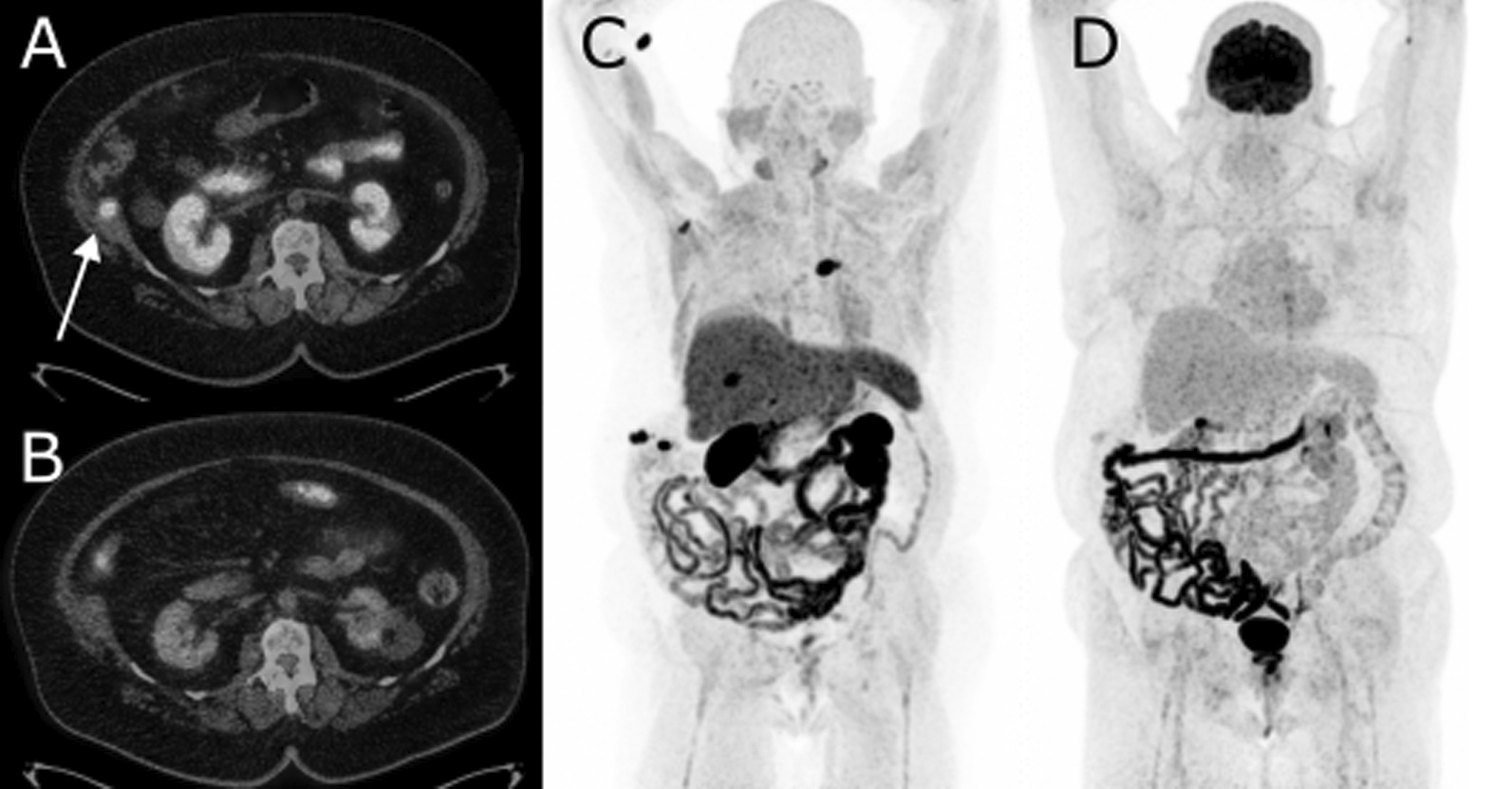
This 75-year-old female patient (#8) with HCC in segment VI and VII, both successfully treated with MWA in 2018 and 2020. Post-treatment MRI follow-up at three months revealed two new lesions in segment V and VIII and several abdominal wall lesions suspected of needle tract metastasis. Dual-tracer PET/CT showed increased uptake of [11C]CH (**A**) for a needle tract site metastasis near the 10th rib (arrow), not seen by [18F]FDG (**B**). In addition, maximum intensity projection (MIP) of [11C]CH (**C**) revealed several intrahepatic metastases and a lesion at fifth costovertebral junction, all not seen on [18F]FDG (**D**). The costovertebral lesion resulted rapidly into spinal cord injury to which patient received emergency radiotherapy. Patient received palliative pain care and died shortly after

**Fig. 2:**
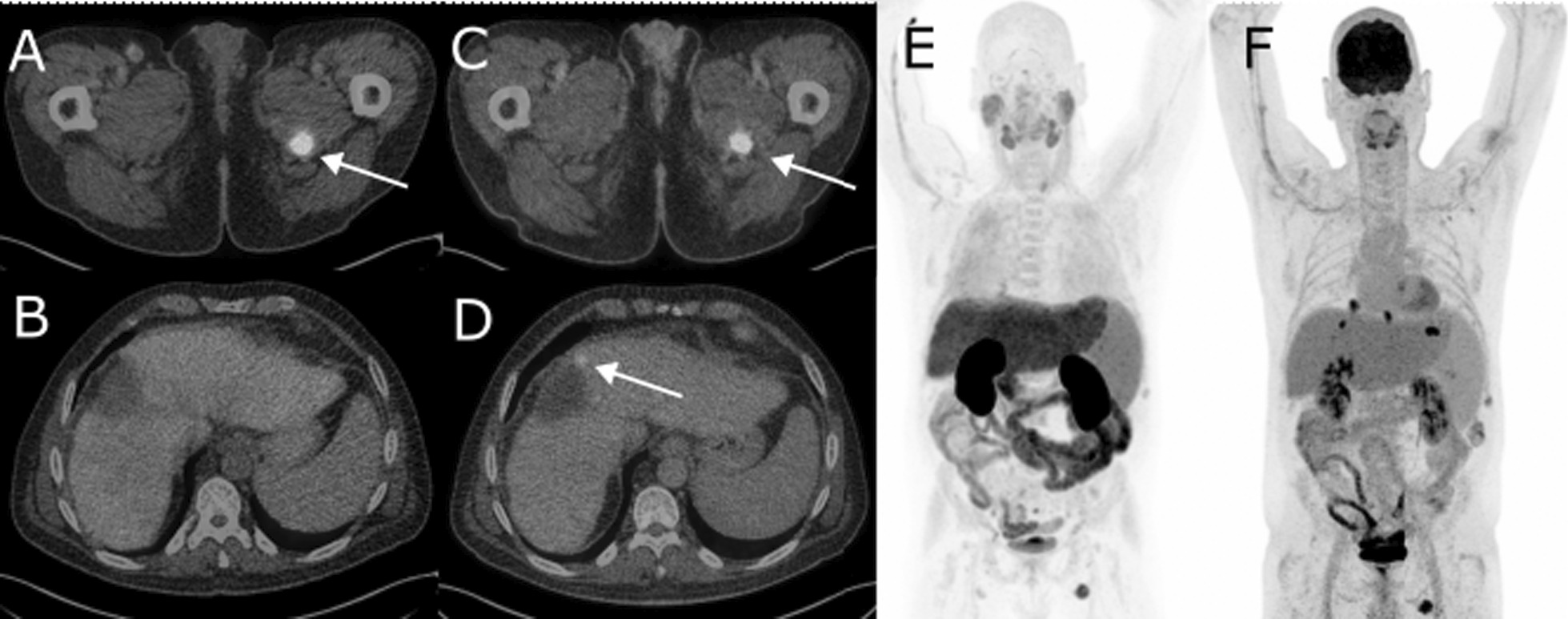
PET/CT imaging of a 49-year-old male patient (#1) known with hemochromatosis-related cirrhosis and multifocal HCC. Underwent MWA of solitary HCC node (32 mm) in segment VIII to ensure eligibility for orthotopic liver transplantation (OLT). Post-therapy MRI follow-up at three months displayed further progression of several intrahepatic lesions and potential tumour thrombus in v. porta, which prompted dual-tracer diagnostic with [18F]FDG and [11C]CH PET/CT. Both tracers found a metastasis in the m. adductor longus muscle (**A** for [11C]CH, and **C** for [18F]FDG, arrow), while an ablation site recurrence was only found by [18F]FDG (**D**) and not by [11C]CH (**B**). In addition, MIP images show several mediastinal lymph nodes only found by [18F]FDG (**F**), whereas [11C]CH (**E**) has physiological uptake in the same region. Due to lymphatic and extrahepatic disease patient received palliative chemotherapy

Intrahepatic HCC recurrence was diagnosed in 65% of all cases by both tracers. One case of vascular involvement by means of a tumour thrombus of the portal vein was confirmed by [^18^F]FDG PET/CT only. Thirteen extrahepatic mHCC lesions were found of which eight lymph nodes with a detected rate of 75% for both tracers. Four soft-tissue lesions were found, all considered mHCC: m. abductor longus (by both PET tracers), pararectal fat (CH-only), thoracic wall (FDG-only), and lung (FDG-only). For therapy-related lesions, two needle tract metastases were found (CH-only, Fig. 1) and 80% of ablation site recurrences were identified by [^18^F]FDG (4/5, Fig. 2) and 40% by [^11^C]CH (2/5). Two cases of novel pathology-proven malignancy were seen: one case of prostate carcinoma (Gleason score 8, CH-only) and one case of bilateral lung carcinoma (both tracers).

After both PET/CT imaging studies, three patients were not considered for treatment and received best supportive care. All other patients received therapy after dual-tracer PET/CT examinations, with among them ten patients undergoing liver-directed therapy and five being treated with systemic therapy (chemotherapy and immunotherapy). A CH-only lesion in the pararectal fat was surgically resected in one patient. Histopathology was available for nine patients and [^11^C]CH detected critical findings in all cases. Both a needle tract metastasis in well-differentiated HCC and progression of a known primary moderately differentiated HCC lesion showed no [^18^F]FDG uptake.

## Discussion

This study found detection rates of 68% for [^18^F]FDG and 84% for [^11^C]CH for lesions that were pivotal in subsequent clinical decision making in patients with HCC. In only one patient both PET tracers failed to detect the anatomical substrate for suspected disease progression, resulting in a combined radiotracer detection rate of 95%. We contend that dual-tracer PET scanning may not have been necessary for this particular patient, given that the lesion was detected through an MRI conducted later, after the dual-tracer PET had already been performed. These promising results underline the potential critical role that dual-tracer approach can have in patients with suspected progressive disease. In the current study, we identified more clinically relevant lesions when combining [^11^C]CH and [^18^F]FDG PET/CT in HCC, compared to [^18^F]FDG or [^11^C]CH alone.

[^18^F]FDG appears to have low sensitivity (27–70%) for intrahepatic HCC, probably due to high background uptake of [^18^F]FDG in liver parenchyma [2, 10]. One study reported a sensitivity rate for the detection of intrahepatic HCC by choline-radiotracer of 88% [22]. Our data shows moderate intrahepatic detection rates for both tracers (65%). Differences in patient selection could explain this: seven out of 19 patients in our study had a direct therapy-related critical finding, such as needle tract metastases and ablation site recurrences. [^18^F]FDG found most ablation site recurrences (80%) and none of the needle tract metastases, with contrasted results for [^11^C]CH (40% and 100%, respectively). HCC tumour aggressiveness, besides technical failure of tumour ablation procedure, is linked to lesions that require multiple bouts of locoregional therapy, of which ablation site recurrence is indicative as well [23]. Our study did not have enough histopathological data to match these results to tumour differentiation.

While both tracers detected a bilateral lung carcinoma, only [^11^C]CH revealed one case of prostate carcinoma (Gleason 8). Detection of extrahepatic metastasis is known to be an independent predictor of poor survival and thus critical in deciding optimal treatment, especially for OLT and liver resections work-up [24, 25]. In four cases out of 16, [^18^F]FDG missed crucial extrahepatic metastases, of which three were identified after addition of [^11^C]CH. Therefore, [^11^C]CH PET/CT scanning in patients with negative extrahepatic [^18^F]FDG scans may increase sensitivity in extrahepatic HCC and suspected needle-tract or local recurrence after local–regional treatment.

Lack of [^18^F]FDG PET/CT uptake in well-differentiated tumours may be due to low amounts of FDG-6-phosphatase activity in these tumour cells [11]. Choline-tracers have shown increased uptake in well-differentiated tumours as these have increased cell membrane metabolism, of which choline is a substrate [18]. It is assumed that both tracers reveal different stages of tumour differentiation, advocating for dual-tracer diagnostics [11]. Although supported by recent meta-analysis, it recommends caution in accepting this rationale, as both methodological differences and lack of standardized histological grading practices were noted [26]. [^18^F]FDG failed to identify one well- and one moderately differentiated tumour in our study: growth of known HCC lesion and a needle tract recurrence. [^11^C]CH identified all lesions with known differentiation. A link between elevated AFP and vascular invasion with [^18^F]FDG avidity has been proposed [26, 27]. Our study found eight cases with elevated AFP or DGCP as reason for dual-tracer PET/CT. One of these lesions was only found by [^18^F]FDG, three only by [^11^C]CH, and four lesions by both tracers. With our limited number of patients, our study does not support a link between differentiation grade or serum tumour marker elevation to [^18^F]FDG or [^11^C]CH detection rates.

Multimodal dual-tracer PET-based imaging can play a significant role in revealing changes in within-tumour metabolism, although the link between oncological changes and metabolic effects are not yet fully understood. Whereas [^18^F]FDG is sensitive for unexplained rise of serum levels of AFP, choline tracers appear to be not [20, 22, 28]. Studies on unexplained serum AFP elevation following locoregional HCC treatment found that [^18^F]FDG PET/CT had detection rates of 64% to 98% [2, 29]. Of the eight patients with elevated tumour markers, seven critical lesions were detected by [^11^C]CH and five by [^18^F]FDG. In all but one the finding was intrahepatic, often growth of a known tumour. Due to heterogeneity in metabolic and/or genetic traits within a single tumour and between lesions within the same patient, overlap between both tracers in their detection rates is plausible [28].

To our knowledge, the presented study is the first to look at intra- and extrahepatic distribution of HCC with a dual-tracer approach with an emphasis on post-therapy follow-up. The retrospective nature, small patient sample, and the limited amount of available histopathology are limiting our conclusions. Further studies combining histopathology, serum tumour markers, four-phase CT/MR imaging, and genetic analysis are essential to further unravel the link between HCC pathogenesis and appropriate detection techniques, such as dual-tracer PET/CT solutions.

## Conclusions

Although PET imaging combining both [^18^F]FDG and Choline-radiotracers has shown its benefit in staging and therapeutic management of patients with HCC, its use is still not commonplace. Due to the intrinsic characteristics of HCC pathogenesis and the resulting metabolic differences, a negative [^18^F]FDG PET/CT does not exclude recurrent HCC. Our study favours a dual-tracer approach in HCC staging when conventional imaging is non-conclusive.

## Data Availability

The datasets used and/or analysed during the current study are available from the corresponding author on reasonable request.

## Abbreviations

BCLC: Barcelona-Clinic Liver Cancer
TACE: Transarterial chemoembolization
SIRT: Selective internal radiotherapy
[^18^F]FDG: [^18^F]-fluoro-2-deoxy-d-glucose
[^11^C]CH: [^11^C]Choline
MWA: Microwave ablation
AFP: Alpha-fetoprotein
DGCP: Des-gamma-carboxy-prothrombin
SABR: Stereotactic ablative radiotherapy
MIP: Maximum intensity projection

## References

[1] de Zalán CMC, Ruiter SJS, van den Berg AP (2022). Outcomes after primary and repeat thermal ablation of hepatocellular carcinoma with or without liver transplantation. Eur Radiol.

[2] Ali SA, Amin DH, Abdelkhalek YI (2020). Efficiency of whole-body [18F]FDG PET CT in detecting the cause of rising serum AFP level in post-therapeutic follow-up for HCC patients. Jpn J Radiol.

[3] Colecchia A, Schiumerini R, Cucchetti A (2014). Prognostic factors for hepatocellular carcinoma recurrence. World J Gastroenterol.

[4] Vogel A, Cervantes A, Chau I (2018). Hepatocellular carcinoma: ESMO Clinical Practice Guidelines for diagnosis, treatment and follow-up. Ann Oncol.

[5] Reig M, Forner A, Rimola J (2022). BCLC strategy for prognosis prediction and treatment recommendation: the 2022 update. J Hepatol.

[6] Mendiratta-Lala M, Masch WR, Shampain K (2020). Mri assessment of hepatocellular carcinoma after localregional therapy: a comprehensive review. Radiol Imaging Cancer.

[7] Imamura H, Matsuyama Y, Tanaka E (2003). Risk factors contributing to early and late phase intrahepatic recurrence of hepatocellular carcinoma after hepatectomy. J Hepatol.

[8] Sharma B, Martin A, Zerizer I (2013). Positron emission tomography-computed tomography in liver imaging. Semin Ultrasound.

[9] Kim S-JJ, Pak K, Koo PJ (2015). The efficacy of (177)Lu-labelled peptide receptor radionuclide therapy in patients with neuroendocrine tumours: a meta-analysis. Eur J Nucl Med Mol Imaging.

[10] Lee SM, Kim HS, Lee S (2019). Emerging role of 18F-fluorodeoxyglucose positron emission tomography for guiding management of hepatocellular carcinoma. World J Gastroenterol.

[11] Bertagna F, Bertoli M, Bosio G (2014). Diagnostic role of radiolabelled choline PET or PET/CT in hepatocellular carcinoma: a systematic review and meta-analysis. Hepatol Int.

[12] Kornberg A, Friess H (2019). 18F-fludeoxyglucose positron emission tomography for diagnosis of HCC: implications for therapeutic strategy in curative and non-curative approaches. Therap Adv Gastroenterol.

[13] Ando E, Tanaka M, Yamashita F (2003). Diagnostic clues for recurrent hepatocellular carcinoma: comparison of tumour markers and imaging studies. Eur J Gastroenterol Hepatol.

[14] Chen WT, Chau GY, Lui WY (2004). Recurrent hepatocellular carcinoma after hepatic resection: prognostic factors and long-term outcome. Eur J Surg Oncol.

[15] Podo F (1999). Tumour phospholipid metabolism. NMR Biomed.

[16] Delbeke D, Martin WH, Sandler MP (1998). Evaluation of benign vs malignant hepatic lesions with positron emission tomography. Arch Surg Arch Surg.

[17] Torizuka T, Tamaki N, Inokuma T (1995). In Vivo assessment of glucose metabolism in hepatocellular carcinoma with FDG-PET. J Nucl Med.

[18] Talbot JN, Michaud L, Grange JD (2014). Use of choline PET for studying hepatocellular carcinoma. Clin Transl Imaging.

[19] Yamamoto Y, Nishiyama Y, Kameyama R (2008). Detection of hepatocellular carcinoma using [11C]CHoline PET: comparison with [18F]FDG PET. J Nucl Med.

[20] Talbot JN, Fartoux L, Balogova S (2010). Detection of hepatocellular carcinoma with PET/CT: A prospective comparison of 18F-fluorocholine and [18F]FDG in patients with cirrhosis or chronic liver disease. J Nucl Med.

[21] Alnammi M, Wortman J, Therrien J (2022). MRI features of treated hepatocellular carcinoma following locoregional therapy: a pictorial review. Abdom Radiol.

[22] Bieze M, Klümpen HJ, Verheij J (2014). Diagnostic accuracy of 18F-methylcholine positron emission tomography/computed tomography for intra- and extrahepatic hepatocellular carcinoma. Hepatology.

[23] Dinorcia J, Florman SS, Haydel B (2020). Pathologic response to pretransplant locoregional therapy is predictive of patient outcome after liver transplantation for hepatocellular carcinoma: analysis from the US multicenter HCC transplant consortium. Ann Surg.

[24] Calvet X, Bruix J, Ginés P (1990). Prognostic factors of hepatocellular carcinoma in the west: a multivariate analysis in 206 patients. Hepatology.

[25] Sneag DB, Krajewski K, Giardino A (2011). Extrahepatic spread of hepatocellular carcinoma: spectrum of imaging findings. Am J Roentgenol.

[26] Ghidaglia J, Golse N, Pascale A (2022). [18F]FDG /18F-choline dual-tracer PET behavior and tumor differentiation in hepatocellular carcinoma. A systematic review. Front Med.

[27] Trojan J, Schroeder O, Raedle J (1999). Fluorine-18 FDG positron emission tomography for imaging of hepatocellular carcinoma. Am J Gastroenterol.

[28] Gougelet A, Sartor C, Senni N (2019). Hepatocellular carcinomas with mutational activation of beta-catenin require choline and can be detected by positron emission tomography. Gastroenterology.

[29] Han AR, Gwak GY, Choi MS (2009). The clinical value of [18F]FDG PET/CT for investigating unexplained serum AFP elevation following interventional therapy for hepatocellular carcinoma. Hepatogastroenterology.

